# "All Sorts and Conditions" of Women

**Published:** 1889-01-26

**Authors:** 


### The Doctor's Pulpit.

### "ALL SORTS AND CONDITIONS" OF WOMEN.

### The Mother of Children.

It seems a very commonplace thing to be a mother, and yet no power possessed by human speech can even approximately describe the thoughts, the feelings, the joys, the pains, the hopes, the anxieties, that gather in the heart of a true woman when she first beholds her newborn child. The love of human parents for their offspring has in it every possibility of the most intense joy on the one hand, and of the most exquisite anguish on the other. That love is felt by both parents, but as a rule most deeply and all-absorbingly by the mother. It is not unnatural that, in early infancy at any rate, the mother's soul should be given up exclusively to her child. The child itself, when it is understood as a mother understands it, has a power of developing and intensifying love in the maternal heart which is as incomprehensible as it is indescribable. A true mother can know no pleasure so pure and deep, no delight so keen and all-pervading as that produced by the glad light of pleased recognition which shines from her little one's eyes. Such is the fulness and intensity of maternal love in the human kind that most mothers, if put to the proof, would lay down their lives for their offspring. It is to mothers of this kind, and on the basis of these admitted facts, that we venture to address a few words from the physiological and health point of view.

It is a very trite thing to say that a woman before the birth of her child should be in the most vigorous condition of health that is possible to her. But though it is trite, it is neither less true nor less important on that account. The truth is that as a rule the commonest things are the most important. It is a very common thing for the sun to shine in summer. When the uncommon happens, and the sun does not shine, that means thin and crude crops for the farmer, and consequent poverty. Therefore, let no woman pass by as unimportant the fundamental statement that before the birth of her child no pains or trouble should be spared to bring her into perfect health. It is necessary both for the mother's and the child's sake; but just now we are emphasising the proposition for the sake of the child. For in many very essential particulars the child will be what the parents are. This is inevitable. There is no fact in any department of science better established than this: that as are the parents, such more or less will be the children. The doctrine of heredity is as true and certain as the doctrine of "free will" or of "Cogito, ergo sum."

What would a mother bestow upon her infant child if fate should give her the power to make her wishes actual? Would she not make him healthy without a flaw, beautiful without the least trace of fault, virtuous as the most unspotted, happy as the most tranquil, prosperous as the hero of a fairy tale? She cannot do all these things for him, but she can do so much that many of them may hereafter be largely accomplished in his history. For instance, she can, if she be healthy herself, give him much of the soundness of her own constitution. Mothers, whose physiological education is nil, may not consider this a point of prime importance; but during the years of infancy, at any rate, health is of much more importance to the human creature than rank or wealth. What does the crowing baby in his nurse's arms care about his parents' station or means? Is he well? Does he enjoy his food, his bath, his daily tours in the open air, his toys, his kittens, and his sleep? If he does, he recks naught of the circumstance whether his father is the duke or the duke's gardener; whether he shall inherit a million when he comes of age, or only sufficient to set him up with a horse and cart as a greengrocer. Health is to him the one prime essential and condition of perfect happiness. Health the mother can often make sure of for him, by keeping herself in a pure and perfectly healthy condition before and after his birth.

But the value of a sound and wholesome constitution is not exhausted with infancy. What a terror it is to the mother of a growing boy or girl to know that there is marked consumption in the family on either side! The smallest sore throat, the slightest cold in the chest, the least loss of appetite or sign of weariness pierces her heart as with a knife. Perhaps no day ever passes without the spectre of a consumptive child, or young man, or young woman presenting itself to her forward-looking imagination. To a sensitive mother forebodings of this kind mingle all life's joys with pain and haunting dread. Her child is bright and joyous now; before he reaches manhood, he may fade away before her eyes into the chilling shadows of an early death. In a lesser degree the inheritance of a tendency to asthma, or rheumatism, or gout, or cancer, or some defect in the sight or hearing, or the like, may be a very serious disadvantage to boy or girl. Such a tendency, developing itself under unfavourable circumstances, may be the one thing which entirely and absolutely mars an otherwise prosperous career, or effectually destroys an otherwise happy life. An attack of rheumatic fever in early youth, for example, may leave an affection of the heart which is never recovered from, and which makes a young man old at a stroke. Similarly a tendency to inherited asthma may show itself in youth, and make a man breathless and helpless when he most wants to be active and robust.

But the limits of heredity are not confined to mere physical manifestations. A child may be a born coward, or a born liar, or a born cheat, or a born fury, or a born fool. Any of these would be terrible. Is the poor mother responsible in such a case? The parents, at any rate, are to some extent; but the mother may be the one on whom the larger responsibility rests. Are there no frivolous young women who remain frivolous after marriage? Do no women contract the habit of lying or deceiving others? Have no women ungovernable tempers? Are no women hopeless fools? Many a woman will enter upon married life with as little sense of responsibility as if she were going to the Lyceum or to Ascot. Such women appear to have no more intellect than a chimpanzee or a dog. Frivolity and inconsequence are the only two distinguishing features of their character. To look to them for sense, or candour, or honesty, or truthfulness, would be as wise as to look to the summit of the Matterhorn for orange trees or vines. Yet these creatures become the mothers of children. Mothers do they call themselves? Physically they are mothers; but intellectually and morally they may as properly be called poets and philosophers as mothers. The only real function of such women is to serve as milliners' models, or as money squanderers to men who are as hopeless fools as themselves.

What function can be greater than that of motherhood? Is it more important to teach a boy or girl how to speak a dead or a living language? Is it worthier to write a second or third rate novel? Is a cookery-book or a series of lectures on sick nursing a higher act than the bringing forth and rearing a healthy living body and a thinking brain? The world is turned topsy-turvy in many people's minds. They have not the slightest idea of the comparative importance of different things. To some mothers an evening party or a new gown infinitely transcends the advent of a new baby, or even the death of a bigger child. Happily, not many are so utterly destitute of the ordinary attributes of womankind as these. But it ought to be understood that the one function of woman, which altogether outweighs every other, is that of being a mother of children. On children the world depends, civilisation depends, virtue depends, intellect depends, everything depends. Thoroughly healthy, thoroughly intelligent, thoroughly conscientious, and thoroughly capable mothers can do more to advance the real prosperity and happiness of the world than Bacon's "Novum Organum," Spencer's "Sociology," or Huxley's "Physiology" or Philosophy. When a healthy woman marries and brings up a family of healthy, honest, and capable children, she does more for the State and for the world than she could do in any other way whatsoever. To despise marriage or seek other ways of life for healthy women is to declare that the order of creation is a mistake, and that Eternal Providence bungled in the making of man.

*(To be continued.)*

